# Biocompatible fluorescent supramolecular nanofibrous hydrogel for long-term cell tracking and tumor imaging applications

**DOI:** 10.1038/srep16680

**Published:** 2015-11-17

**Authors:** Huaimin Wang, Duo Mao, Youzhi Wang, Kai Wang, Xiaoyong Yi, Deling Kong, Zhimou Yang, Qian Liu, Dan Ding

**Affiliations:** 1 State Key Laboratory of Medicinal Chemical Biology, Key Laboratory of Bioactive Materials, Ministry of Education, College of Life Sciences, Nankai University, Tianjin, 300071, P. R. China; 2 Collaborative Innovation Center of Chemical Science and Engineering (Tianjin), Nankai University, Tianjin, 300071, P. R. China; 3 Department of Urology, Tianjin First Central Hospital, 24 Fukang Road, Tianjin 300192, P. R. China

## Abstract

Biocompatible peptide-based supramolecular hydrogel has recently emerged as a new and promising system for biomedical applications. In this work, Rhodamine B is employed as a new capping group of self-assembling peptide, which not only provides the driving force for supramolecular nanofibrous hydrogel formation, but also endows the hydrogel with intrinsic fluroescence signal, allowing for various bioimaging applications. The fluorescent peptide nanofibrous hydrogel can be formed *via* disulfide bond reduction. After dilution of the hydrogel with aqueous solution, the fluorescent nanofiber suspension can be obtained. The resultant nanofibers are able to be internalized by the cancer cells and effectively track the HeLa cells for as long as 7 passages. Using a tumor-bearing mouse model, it is also demonstrated that the fluorescent supramolecular nanofibers can serve as an efficient probe for tumor imaging in a high-contrast manner.

Supramolecular hydrogel formed by gelator and water *via* non-covalent interactions is attracting considerable interest in many a research area including tissue engineering^1^,^2^,^3^,^4^,^5^, analyte detecting^6^,^7^,^8^,^9^, three-dimensional (3D) cell culture^10^,^11^,^12^, drug delivery^13^,^14^,^15^,^16^,^17^, wound healing^18^,^19^, immune responding^20^ as well as inhibitor screening^21^. In general, the formed supramolecular hydrogel consists of micrometer- or nanometer-length nanofibers that are entangled with each other^22^. Among various supramolecular hydrogels, peptide-based hydrogel through self-assembly has shown a unique merit in biomedical applications by virtue of their ease design and modification, inherent biocompatibility, large drug carrying capacity, low immunity and good adaptability to multiple functions^23^,^24^. The nanofibrous hydrogel of peptides often spontaneously form with non-covalent interactions such as π-π stacking, hydrophobic interaction and hydrogen bonding, as the main driving forces for self-assembly^22^. To introduce hydrophobic and π-π interactions in the short peptide-based supramolecular hydrogel, an aromatic capping group is often required. To date, 9-fluorenyl-methoxycarbonyl (Fmoc)^25^ and naphthalene (Nap)^26^,^27^,^28^ have been widely utilized as the aromatic capping groups. Besides, other capping groups including pyrene^29^, perylene^30^, anthracene^31^, indole^32^ and adamantane^33^ have also been established.

In order to push forward the practical biomedical applications of supramolecular hydrogels such as drug delivery and regenerative medicine, the *in vivo* biocompatibility and stability need to be evaluated. Williams and co-workers reported *in vivo* performance of an enzyme-assisted self-assembled peptide/protein hydrogel in the model of zebrafish *via* microinjection into zebrafish muscle^34^; Ghanaati’s group estimated the biocompatibility and stability of supramolecular hydrogel formed by a peptide amphiphile in subcutaneous space^35^; Zhang’s group also assessed the biocompatibility and stability of a Fmoc-short peptide-based supramolecular hydrogel in subconjunctival space and anterior chamber^36^; Our group systematically studied the *in vivo* dynamic biostability, biodistribution and toxicity of both L- and D-amino acid-based self-assembling peptide nanofibers^37^. These studies have explicitly demonstrated the excellent biocompatibility of the self-assembling peptide-based supramolecular hydrogels, which endow them with great promise in biomedical applications and potential clinical translation. Despite of the exciting work in the field of peptide-based supramolecular hydrogel, rather limited work focused on the development of fluorescent supramolecular nanofibers/hydrogels^22^. Very recently, Xu and co-workers introduced 4-nitro-2,1,3-benzoxadiazole (NBD) and Dansyl as the capping group of short peptide, respectively, for imaging and monitoring enzymatic triggered self-assembly inside live cells, offering new insights at the interface of chemistry and biology^38^,^39^,^40^. As the fluorescence technique provides scientists with sights and insights into both the physiological and pathological processes in a real-time and sensitive manner^41^,^42^,^43^, exploration of novel fluorescent peptide-based supramolecular hydrogel with the combined advantages of fluorescence and supramolecular hydrogel is highly desirable for biomedical applications.

In this contribution, we report for the first time that Rhodamine B is employed as a new capping group of self-assembling peptide to provide the hydrophobic and π-π interactions for hydrogel formation. A precursor peptide of Rhodamine-GFFYE-CS-EE was synthesized and a biocompatible strategy of disulfide bond reduction was used to facilitate the formation of fluorescent supramolecular nanofibrous hydrogel. After dilution of the hydrogel with aqueous solution, the fluorescent nanofiber suspension can be obtained, which has given a superb performance in long-term cell tracking and tumor imaging applications. This study thus offers fundamental guidelines to design and yield fluorescent supramolecular nanofibrous hydrogel, which will also inspire more exciting research in developing novel nanofibrous hydrogel-based fluorescent probes for a variety of bioimaging applications.

## Results

### Synthesis and characterization of the precursor and supramolecular nanofibrous hydrogel

**Figure 1:**
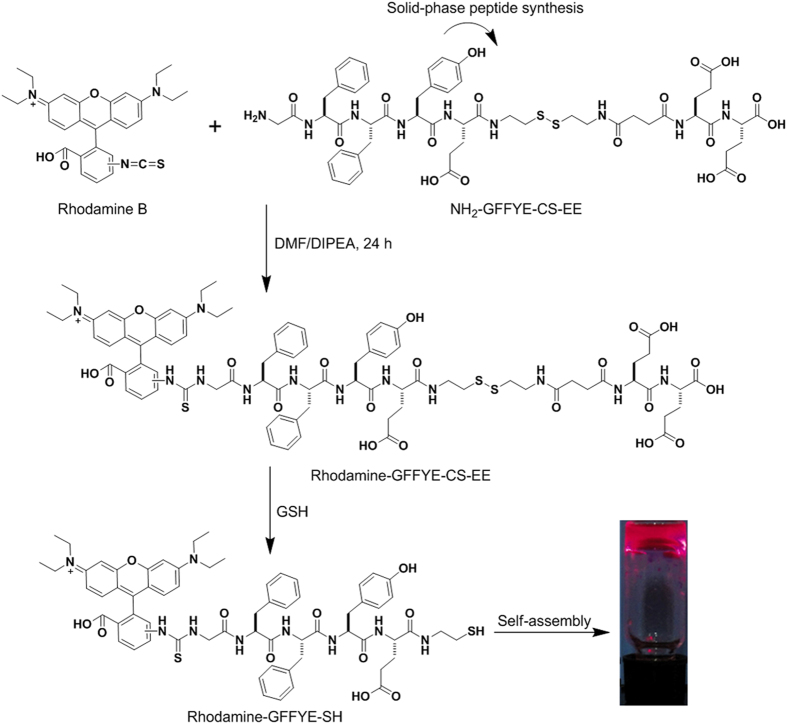
The compound synthesis and formation of supramolecular nanofibrous hydrogel. Synthetic route to Rhodamine-GFFYE-CS-EE and its self-assembly to supramolecular hydrogel *via* the reduction of glutathione (GSH). The photograph of red fluorescent hydrogel was taken under illumination of a UV lamp.

The chemical structure of the peptide of Rhodamine-GFFYE-CS-EE is shown in Fig. 1. NH_2_-GFFYE-CS-EE was firstly synthesized by standard Fmoc solid-phase peptide synthesis (SPPS). Subsequent coupling between Rhodamine B isothiocyanate and NH_2_-GFFYE-CS-EE afforded Rhodamine-GFFYE-CS-EE in 80% yield. The product was characterized by ^1^H NMR and LC-MS (Supplementary Fig. S1 and S2). The design rationale of Rhodamine-GFFYE-CS-EE is as follows: 1) Rhodamine B, a well-known fluorogen with long-wavelength absorption and emission as well as high quantum efficiency, has been widely utilized in the field of bioimaging and biosensing^44^. In this study, the introduction of Rhodamine B as the capping group of self-assembling peptide not only supports the driving force for self-assembly, but also offers a new opportunity to track the fate of the resulting nanofibers/hydrogel in various biomedical applications by taking advantage of its high fluorescence signal; 2) the hydrophilic EE (E: glutamic acid) sequence at the other end of molecule can endow Rhodamine-GFFYE-CS-EE with good water-solubility. Rhodamine-GFFYE-CS-EE thus serves as the precursor of the supramolecular hydrogel; 3) the tetra-peptide GFFY has been demonstrated to possess superb self-assembly property and gelation ability^45^; 4) the cystamine succinate (CS) part in the peptide includes a disulfide bond, which is able to be cleaved by reductants (*e.g.*, glutathione (GSH))^46^. After removal of the hydrophilic EE, it is hypothesized that the residue peptide will self-assemble into homogeneous supramolecular hydrogel.

**Figure 2:**
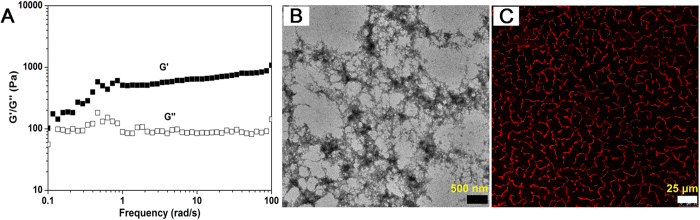
Characterization of the nanofibrous hydrogel. (**A**) Frequency sweep, (**B**) TEM image and (**C**) CLSM image of the hydrogel formed by Rhodamine-GFFYE-CS-EE at the concentration of 0.5 wt%.

To test our hypothesis, the gelation ability of Rhodamine-GFFYE-CS-EE in the presence of reductant was assessed by a vial inversion method. As shown in Fig. 1, red fluorescent hydrogel was successfully formed after addition of GSH at room temperature for 2 h. Noteworthy, the gelation process would be much faster in around 30 min when the temperature elevates to 37 ^o^C. The HPLC result confirms the cleavage of disulfide bond in the Rhodamine-GFFYE-CS-EE upon reaction with GSH (Supplementary Fig. S3). The viscoelasticity is one of the main characteristic properties of the hydrogel, which indicates the mechanical property of the hydrogel. Therefore, rheological measurement of the hydrogel was carried out. As shown in Fig. 2A, frequency sweep at constant oscillation amplitude (1.0%) provides us information on the Rhodamine-GFFYE-CS-EE after addition of GSH. The storage modulus (G’) is several times larger than the loss modulus (G”), suggesting the formation of a typical supramolecular hydrogel. Transmission electron microscopy (TEM) result reveals the filamentous network structures in the hydrogel (Fig. 2B). By virtue of the high emission of Rhodamine B, it is convenient for us to investigate the nanostructures in the hydrogel through confocal laser scanning microscopy (CLSM). The CLSM observation indicates homogeneous fluorescent nanofibers with length of several micrometers. Supplementary Fig. S4 shows the UV-vis absorption and photoluminescence (PL) spectra of the resultant nanofibers in water (The hydrogel was diluted with water, leading to nanofiber suspension). The red fluorescence with an emission maximum of 585 nm makes the nanofibers/hydrogel very promising for bioimaging application with low interferential absorption and background fluorescence^47^.

### Cellular uptake and long-term cell tracking

**Figure 3:**
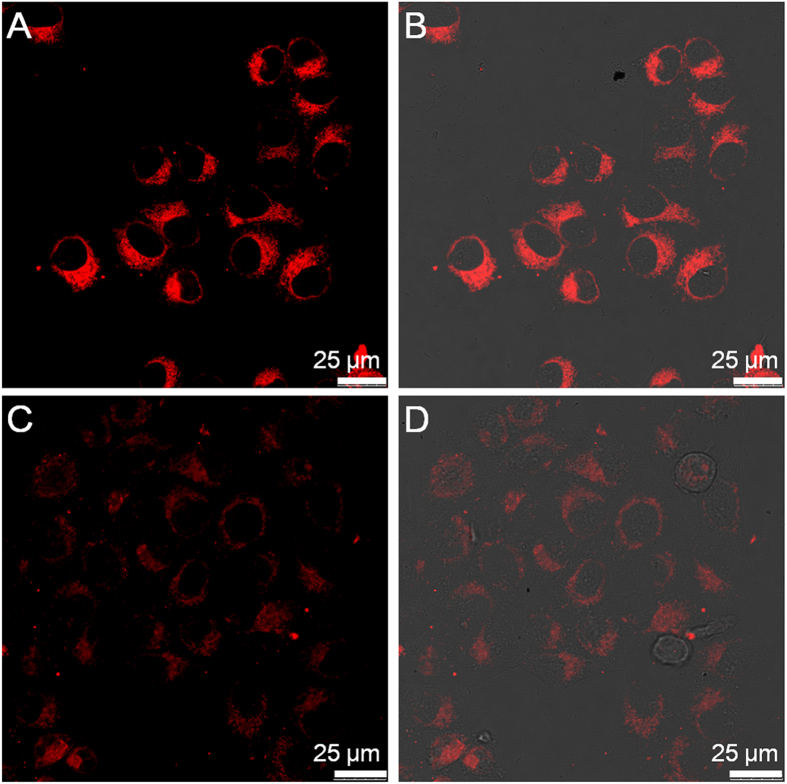
Cellular uptake study. CLSM images of the HeLa cancer cells after incubation with (**A**) the nanofiber and (**C**) precursor for 2 h. (**B**) and (**D**) are the corresponding fluorescence/transmission overlay images of (**A**) and (**C**), respectively.

The cellular uptake of the precursor and nanofiber as well as their spatial distribution in cells were then investigated with CLSM. After hydrogel formation by addition of GSH, the nanofiber suspension was obtained by dilution of the hydrogel with cell culture medium. Fig. 3 displays the CLSM images of the HeLa cancer cells after incubation with the precursor and nanofiber at the Rhodamine B concentration of 100 μM for 2 h, respectively. It can be seen that both the precursor and nanofiber are internalized in the cell cytoplasm. Additionally, it is obvious that the fluorescence intensity of nanofiber-treated HeLa cells (Fig. 3A) is far higher as compared to that of precursor-treated cells (Fig. 3C). Similar result was also observed when the HeLa cells were changed to HepG2 cancer cells (Supplementary Fig. S5). Noteworthy, at the same Rhodamine B concentration, the fluorescence intensity of nanofiber is around 1.6 times lower than that of precursor owing to the aggregation caused quenching effect of the small-molecule organic dye (Supplementary Fig. S6)^48^. The higher fluorescence intensity of nanofiber-treated cells should be due to the higher cellular uptake efficiency of nanofibers through endocytosis^49^. It is noted that there is no detectable autofluorescence from the untreated cancer cells under the same imaging conditions (Supplementary Fig. S7). These results suggest that as compared to the small molecular precursor, the nanofiber formulation can favour more molecules enter into the cells. Moreover, the low cytotoxicity of the precursor and nanofiber encourages us to use them for further bioimaging applications (Supplementary Fig. S8).

**Figure 4:**
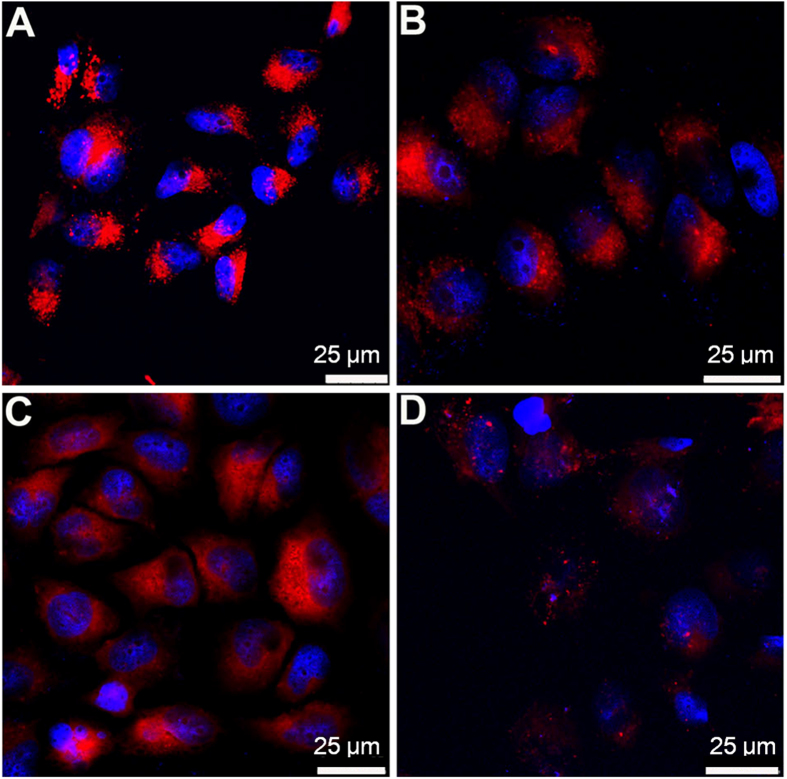
Long-term cell tracing study. CLSM images of the fluorescent nanofiber-stained HeLa cancer cells at different passages. (**A**) 1 passage; (**B**) 3 passages; (**C**) 5 passages; (**D**) 7 passages. The cell nuclei were stained with DAPI.

Long-term cell tracking have recently attracted intensive research interest, as it can provide meritorious information on cell therapy and its processes, as well as on studying the mechanism of cancer pathogenesis, invasion and metastasis^50^. Consequently, the application of the fluorescent nanofibrous hydrogel in long-term cell tracking was investigated. The HeLa cancer cells were incubated with the nanofiber suspension for 4 h at 37 ^o^C. Subsequently, the nanofiber-labeled cells were subcultured for designated time intervals, followed by imaging with CLSM. As shown in Fig. 4, the HeLa cells stained with nanofibers emit bright red fluorescence at the first passage. Although the fluorescence intensity of the nanofiber-stained cells gradually decreases with an increase in the number of passages, our fluorescent nanofibers are still capable of effectively tracking the HeLa cells for as long as 7 passages. It has been established that the best-known commercially available cell tracker, CellTrackerTM Green CMFDA, has the ability to trace the living cells for no more than 3 passages^51^,^52^. Our nanofibers thus hold great promise as an efficient red fluorescent tracker for long-term cell tracing application.

### Tumor imaging application

**Figure 5:**
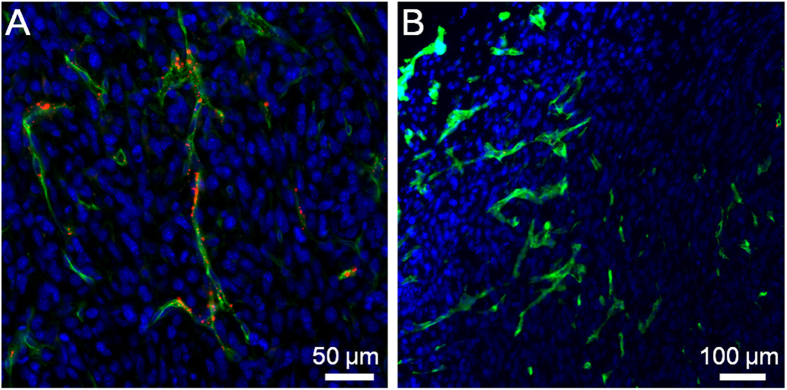
Tumor accumulation of nanofiber and precursor. CLSM images of tumor slices from mice after intravenous injection of (**A**) nanofiber and (**B**) precursor, respectively, for 0.5 h. Tumor blood vessels were immunostained with PECAM-1 (green). The cell nuclei were stained with DAPI.

The previous work by Discher and co-workers demonstrated that the flexible nanofibers exhibited unique advantages over spherical particles (*e.g.*, longer blood circulation time), which render the flexible nanofibers with great promise for drug delivery and bioimaging applications^53^. In this regard, the utility of the precursor and nanofiber in *in vivo* tumor imaging was also studied. To establish the tumor-bearing mouse model, murine 4T1 cancer cells were subcutaneously inoculated into the right axillae of the mice. For fluorescence imaging, the nanofiber and precursor were intravenously administrated into the 4T1 tumor-bearing mice, respectively. At 0.5 h post-injection, both the nanofiber and precursor-treated tumor tissues were sliced and the tumor vasculature was immunostained against platelet/endothelial cell adhesion molecule 1 (PECAM-1)^54^. As displayed in Fig. 5A, many a bright dot with red fluorescence is distributed in and close to the tumor blood vessels (green fluorescence). In comparison, negligible red fluorescence is observed in the slices of precursor-treated tumors (Fig. 5B). This result indicates that as compared to the small molecular precursor, the nanofiber is capable of rapidly accumulating into the tumor mass by the enhanced permeability and retention (EPR) effect by virtue of its nanoscale size^55^.

**Figure 6:**
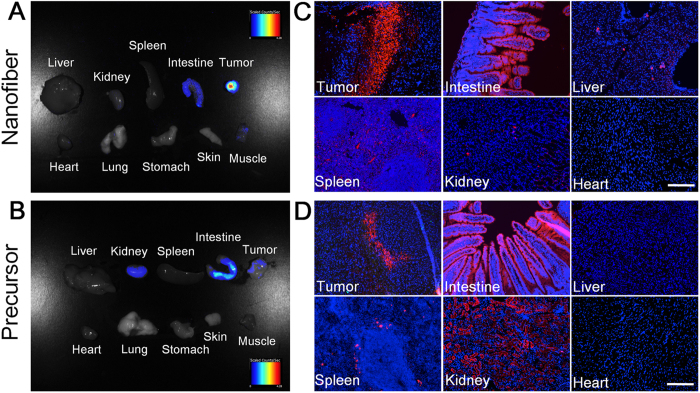
Tumor imaging and biodistribution of nanofiber and precursor. *Ex vivo* fluorescence imaging of tumor tissue and major organs of mice after intravenously injection of (**A**) nanofiber and (**B**) precursor. Fluorescence images of tissue slices from mice treated with (**C**) nanofiber and (**D**) precursor. The mice were sacrificed at 24 h post-injection. For (**C**) and (**D**), the cell nuclei were stained with DAPI. Scale bar: 200 μm for all the fluorescence images in (**C**) and (**D**).

Furthermore, at 24 h post-administration, the mice were sacrificed and the tumor tissues as well as main organs were collected for imaging using a Maestro EX fluorescence imaging system. Figure 6A,B display the fluorescence images of various tissues from 4T1 tumor-bearing mice treated with nanofiber and precursor, respectively. As shown in Fig. 6A, strong fluorescence signal is observed in tumor, verifying the prominent EPR effect of the nanofibers. In addition, relatively weak fluorescence signal can also be detected in the mouse intestine. In contrast, as shown in Fig. 6B, obvious fluorescence signals are located in the tissues of tumor, intestine and kidney, indicating that the precursor biodistribution in tumor-bearing mice is different from that of the nanofibers. Noteworthy is that the fluorescence intensity in the precursor-treated tumors is far weaker than that in nanofiber-treated tumors, substantiating the superior tumor targeting capability of the nanofibers. Subsequently, the tissues were sliced and the nanofiber (Fig. 6C) and precursor (Fig. 6D) distributions in these tissues were studied at cellular resolution as well. As compared to the precursor-treated tumor slices, much more fluorescent aggregates are observed in the nanofiber-treated tumor slices. Overall, the results in Fig. 6C,D agree very well with those in Fig. 6A,B. These data together reveal that our fluorescent nanofibers can serve as an effective probe for tumor imaging application.

## Discussion

In conclusion, we report a fluorescent supramolecular nanofibrous hydrogel for long-term cell tracking and tumor imaging applications. Rhodamine B is demonstrated for the first time to be a new capping group to provide the hydrophobic and π-π interactions for supramolecular nanofibrous hydrogel formation. The fluorescent nanofiber suspension can be obtained by dilution of the hydrogel with aqueous solution. The resultant nanofibers with low cytotoxicity are able to effectively trace the HeLa cells for as long as 7 passages. The *in vivo* studies reveal that as compared to the precursor, the nanofibers can preferentially accumulate into tumor tissues by EPR effect, allowing for tumor imaging in a high-contrast manner. The red fluorescence makes the nanofibers promising for bioimaging application in terms of relatively low interferential absorption and relatively high tissue penetration.

## Methods

### Chemicals

Fmoc-OSu and other Fmoc-amino acids were obtained from GL Biochem (Shanghai, China). 2-Cl-trityl chloride resin (1.0–1.2 mmol/g) was obtained from Nankai University Resin Co. Ltd. Rhodamine B isothiocyanate (RBITC) was received from Aladdin. Paraformaldehyde was obtained from Beijing Solarbio Science & Technology Co., Ltd. Dulbecco’s modified Eagle’s medium (DMEM), fetal bovine serum (FBS) and penicillin/streptomycin were purchased from Gibco Corporation. All the other chemical reagents and solvents were purchased from Alfa (China) and used as received from commercial sources.

### General methods

^1^H NMR spectra were recorded on a Bruker ARX 400 using DMSO-*d*_6_ as the solvent. High-pressure liquid chromatography (HPLC) was conducted at a LUMTECH HPLC (Germany) system using a C_18_ RP column with MeOH (0.05% of TFA) and water (0.05% of TFA) as the eluents. Liquid chromatography mass spectra (LC-MS) were performed at a LCMS-20AD (Shimadzu) system. UV-vis spectra were measured on a Shimadzu UV-1700 spectrometer. Photoluminescence spectra were measured on a Perkin-Elmer LS 55 spectrofluorometer. The sample morphology was studied by transmission electron microscopy (JEM-2010F, JEOL, Japan). Rheology test was carried out on an AR 1500ex (TA instrument) system, and 40 mm parallel plates was used during the experiment at the gap of 500 μm. For the dynamic frequency sweep, the solution of Rhodamine-GFFYE-CS-EE was directly transferred to the rheometer in the region of 0.1–100 rad/s at the strain of 1%.

### Peptide systhesis

Peptide of NH_2_-GFFYE-CS-EE was synthesized by standard Fmoc solid-phase peptide synthesis (SPPS) using 2-chlorotrityl chloride resin and the corresponding N-Fmoc protected amino acids with side chains properly protected. To synthesize Rhodamine-GFFYE-CS-EE, 20 mg (40 μmol) of RBITC was dissolved in 4 mL of DMF, to which was then added 69 mg of NH_2_-GFFYE-CS-EE (40 μmol). DIPEA was used to adjust the final pH of the mixture to 8–9. After reaction for 24 h at room temperature, the final product was purified by HPLC (50 mg, 80% yield). ^1^H NMR (400 MHz, DMSO-*d*_6_): *δ* 8.34 (d, *J* = 8.8 Hz, 1 H), 8.20–7.91 (m, 6 H), 7.23–7.03 (m, 7 H), 7.10–6.95 (m, 3 H), 6.64 (d, *J* = 8.4 Hz, 2 H), 4.68–4.40 (m, 3 H), 4.30–4.17 (m, 3 H), 4.15–4.01 (m, 1 H), 3.76–3.40 (m, 6 H), 3.38–3.20 (m, 10 H), 3.17–2.82 (m, 3 H), 2.80–2.61 (m, 5 H), 2.41–2.10 (m, 7 H), 2.01–1.60 (m, 6 H), 1.38–0.92 (m, 11 H). MS: *m*/*z* [M + H]^+^ calc. 1656.92, obsvd. 1656.70.

### Nanofibrous hydrogel formation

5.0 mg of Rhodamine-GFFYE-CS-EE and 3 equiv. of Na_2_CO_3_ (in order to adjust the pH value of the final solution) were dissolved in 1.0 mL of PBS buffer (pH 7.4), and then 4 equiv. of reduced glutathione (GSH) was added to the solution. After about 20 minutes at room temperature, the self-supporting hydrogel was formed. Moreover, after dilution of the hydrogel with aqueous solution, the fluorescent nanofiber suspension was obtained.

### Cell culture

Human HeLa and HepG2 cancer cells and murine 4T1 cancer cells were cultured in DMEM containing 10% FBS and 1% penicillin-streptomycin at 37 ^o^C in a humidified environment containing 5% CO_2_, respectively. Before experiments, the cells were pre-cultured until confluence was reached.

### Cellular uptake and imaging

The HeLa cancer cells were cultured in confocal imaging chambers (LAB-TEK, Chambered Coverglass System) at 37 ^o^C. After 80% confluence, the medium was removed and the adherent cells were washed twice with 1 × PBS buffer. The nanofiber and precursor in FBS-free DMEM medium at the Rhodamine B concentration of 100 μM were then added to the chamber, respectively. After incubation for 2 h, the cells were washed with 1 × PBS buffer and then imaged with confocal laser scanning microscope (CLSM, Zeiss LSM 410, Jena, Germany). The fluorescent signal from the Rhodamine B was collected upon excitation at 543 nm with a 560 nm longpass barrier filter. The HepG2 cancer cellular imaging was performed following the same experimental procedures.

### Long-term cell tracking

The HeLa cancer cells were cultured in 6-well plates (Costar, IL, USA) to achieve 80% confluence. After medium removal and washing with 1 × PBS buffer, 100 μM of fluorescent nanofibers in DMEM medium were then added to the wells. After 4 h incubation at 37 ^o^C, the labeled cells were washed twice with 1 × PBS buffer, detached by 1 × tripsin and resuspended in culture medium. Upon dilution, the cells were subcultured in 6-well plates containing cell culture coverslips for 1, 3, 5 and 7 passages, respectively. The cells were subsequently fixed, stained with 4′,6-diamidino-2-phenylindole (DAPI) and imaged with CLSM.

### Cytotoxicity study

The cytotoxicities of the fluorescent nanofiber and precursor against HeLa cells were assessed by 3-(4,5-dimethylthiazol-2-yl)-2,5-diphenyltetrazolium bromide (MTT) assay. In brief, HeLa cells were exposed to nanofiber and precursor in cell culture medium at the Rhodamine B concentration of 100 μM at 37 ^o^C, respectively. To eliminate the UV absorption interference of the samples at 570 nm, the control cells were incubated with nanofiber or precursor at the same concentration. After the designated time intervals, the sample wells were washed twice with 1 × PBS buffer and 100 μL of freshly prepared MTT solution (0.5 mg/mL) in culture medium was added into each sample well. The MTT medium solution was carefully removed after 3 h incubation in the incubator for the sample wells, whereas the control wells without addition of MTT solution were washed twice with 1 × PBS buffer. DMSO (100 μL) was then added into each well and the plate was gently shaken for 10 min at room temperature to dissolve all the precipitates formed. The absorbance of individual wells at 570 nm was then monitored by the microplate Reader (GENios Tecan). The absorbance of MTT in the sample well was determined by the differentiation between the absorbance of the sample well and that of the corresponding control well. Cell viability was expressed by the ratio of the absorbance of MTT in the sample wells to that of the cells incubated with culture medium only.

### Animals and tumor-bearing mouse medel

All animal studies were performed under the guidelines set by the Tianjin Committee of Use and Care of Laboratory Animals, and the overall project protocols were approved by the Animal Ethics Committee of Nankai University. Eight-week-old BALB/c mice were purchase from the Laboratory Animal Center of the Academy of Military Medical Sciences (Beijing, China). To establish tumor-bearing mouse model, a 4T1 cancer cell suspension containing 5 × 10^5^ cells (50 μL) was injected subcutaneously into the mice at the right axillae.

### *Ex vivo* fluorescence imaging

4T1 tumor-bearing mice were intravenously administrated with 0.1 mL of fluorescent nanofiber or precursor (100 μM). At 24 h post-injection, the mice were sacrificed, the tissues including liver, kidney, spleen, intestine, heart, lung, stomach, skin, muscle and tumor were excised and imaged using the Maestro system (CRi, Inc., Woburn, USA). Light with a central wavelength of 523 nm was selected as the excitation source. Spectral imaging from 560 to 900 nm with 10 nm steps was carried out with an exposure time of 150 ms.

### Fluorescence imaging in tissue slices

At 0.5 h post administration of 0.1 mL of fluorescent nanofiber and precursor (100 μM), respectively, the mice were sacrificed. Subsequently, the tumor tissues were dissected, fixed in 4% paraformaldehyde for 2 h, incubated in 20% sucrose/PBS overnight and embedded in Optimal Cutting Temperature (OCT) compound (Tissue-Tek). Sections (6 μm) were immunostained with monoclonal antibody against platelet/endothelial cell adhesion molecule 1 (PECAM-1; PharMingen). Alexa Fluor 633-conjugated antirabbit antibody was used as secondary antibody (Molecular Probes). The cell nuclei were stained with DAPI. The tumor slices were imaged by CLSM with excitation wavelength at 405 nm, 543 nm, and 633 nm for DAPI, Rhodamine B, and PECAM-1, respectively. Furthermore, at 24 h post administration of nanofiber and precursor, respectively, the slices of various tissues including tumor, intestine, liver, spleen, kidney, and heart were obtained following the aforementioned experimental procedures. After that, the cell nuclei in the tissue slices were stained with DAPI, which was followed by imaging with CLSM.

## Additional Information

**How to cite this article**: Wang, H. *et al.* Biocompatible fluorescent supramolecular nanofibrous hydrogel for long-term cell tracking and tumor imaging applications. *Sci. Rep.* **5**, 16680; doi: 10.1038/srep16680 (2015).

## Supplementary Material

Supplementary Information
