# Supplementary material for: Biocompatible fluorescent supramolecular nanofibrous hydrogel for long-term cell tracking and tumor imaging applications
Source: Sci Rep. 2015 Nov 17;5:16680. doi: 10.1038/srep16680 (PMC4647837; doi:10.1038/srep16680)
Supplement: Supplementary Information [file srep16680-s1.doc]

**Supplementary Information**

Biocompatible fluorescent supramolecular nanofibrous hydrogel for long-term cell tracking and tumor imaging applications

Huaimin Wang^1^*, Duo Mao^1^*, Youzhi Wang^1^, Kai Wang^1^, Xiaoyong Yi^2^, Deling Kong^1^, Zhimou Yang^1^, Qian Liu^2^ & Dan Ding^1^

^1^State Key Laboratory of Medicinal Chemical Biology, Key Laboratory of Bioactive Materials, Ministry of Education, College of Life Sciences, and Collaborative Innovation Center of Chemical Science and Engineering (Tianjin), Nankai University, Tianjin, 300071, P. R. China, ^2^Department of Urology, Tianjin First Central Hospital, 24 Fukang Road, Tianjin 300192, P. R. China.

Correspondence and requests for materials should be addressed to D.D. (dingd@nankai.edu.cn) or Q.L. (simonlq@163.com) or K.W. (wkcs424@163.com).

* These authors contributed equally to this work.


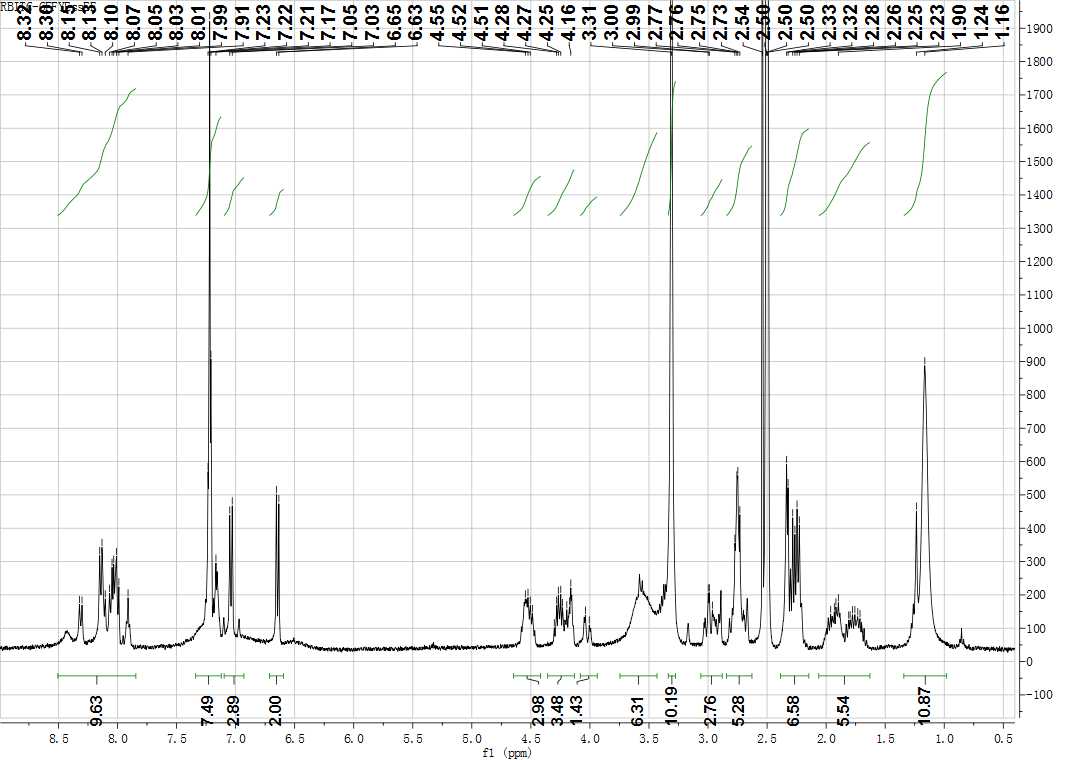


**Supplementary Figure S1** ^1^H NMR spectrum of Rhodamine-GFFYE-CS-EE.

**Supplementary Figure S2** LC-MS spectrum of Rhodamine-GFFYE-CS-EE.


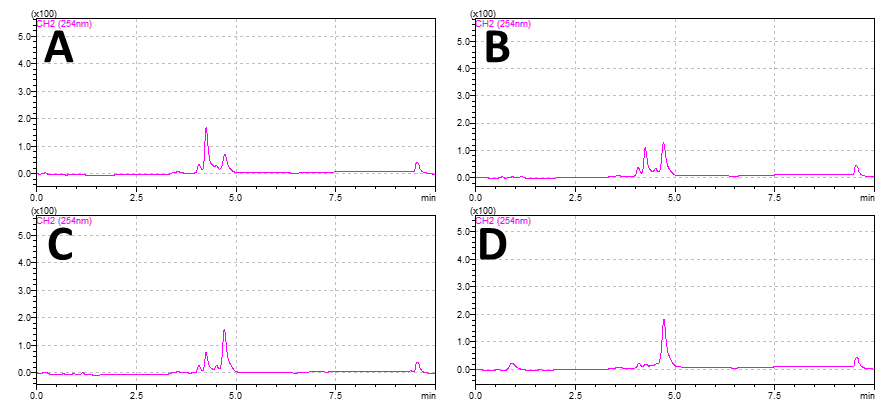


**Supplementary Figure S3** HPLC spectra of Rhodamine-GFFYE-CS-EE after treatment with GSH for (A) 1 h, (B) 2 h, (C) 3 h and (D) 17 h.

**Supplementary Figure S4** UV-vis absorption and photoluminescence spectra of the nanofibers in water. Excitation at 553 nm.

**Supplementary Figure S5** CLSM images of the HepG2 cancer cells after incubation with (A) the nanofiber and (C) precursor for 2 h. (B) and (D) are the corresponding fluorescence/transmission overlay images of (A) and (C), respectively.

**Supplementary Figure S6** Photoluminescence spectra of the precursor and nanofiber in water. Excitation at 553 nm.


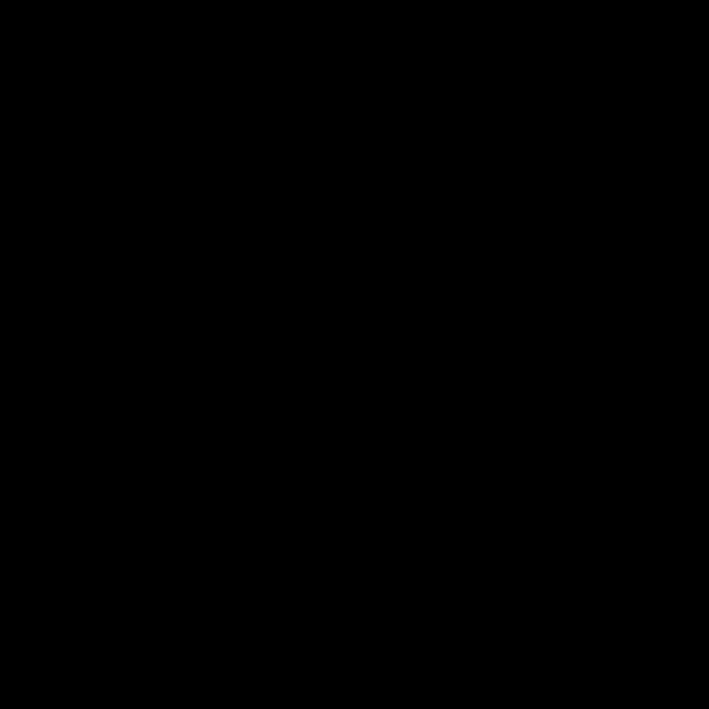


**Supplementary Figure S7** CLSM image of the unlabeled HeLa cancer cells.

**Supplementary Figure S8** Metabolic viability of HeLa cells after incubation with the precursor and nanofiber at the Rhodamine B concentration of 100 μM for 12, 24 and 48 h.
